# Factors Affecting Targeted Sequencing of 353 Nuclear Genes From Herbarium Specimens Spanning the Diversity of Angiosperms

**DOI:** 10.3389/fpls.2019.01102

**Published:** 2019-09-18

**Authors:** Grace E. Brewer, James J. Clarkson, Olivier Maurin, Alexandre R. Zuntini, Vanessa Barber, Sidonie Bellot, Nicola Biggs, Robyn S. Cowan, Nina M. J. Davies, Steven Dodsworth, Sara L. Edwards, Wolf L. Eiserhardt, Niroshini Epitawalage, Sue Frisby, Aurélie Grall, Paul J. Kersey, Lisa Pokorny, Ilia J. Leitch, Félix Forest, William J. Baker

**Affiliations:** ^1^Science Directorate, Royal Botanic Gardens, Kew, Richmond, United Kingdom; ^2^School of Life Sciences, University of Bedfordshire, Luton, Bedfordshire United Kingdom; ^3^Department of Bioscience, Aarhus University, Ny Munkegade Aarhus C, Denmark; ^4^Centre for Plant Biotechnology and Genomics (CBGP, UPM-INIA), Pozuelo de Alarcón, Madrid, Spain

**Keywords:** angiosperms, herbarium specimens, degraded DNA, genomics, high-throughput sequencing, target enrichment, DNA barcoding, herbariomics

## Abstract

The world’s herbaria collectively house millions of diverse plant specimens, including endangered or extinct species and type specimens. Unlocking genetic data from the typically highly degraded DNA obtained from herbarium specimens was difficult until the arrival of high-throughput sequencing approaches, which can be applied to low quantities of severely fragmented DNA. Target enrichment involves using short molecular probes that hybridise and capture genomic regions of interest for high-throughput sequencing. In this study on herbariomics, we used this targeted sequencing approach and the Angiosperms353 universal probe set to recover up to 351 nuclear genes from 435 herbarium specimens that are up to 204 years old and span the breadth of angiosperm diversity. We show that on average 207 genes were successfully retrieved from herbarium specimens, although the mean number of genes retrieved and target enrichment efficiency is significantly higher for silica gel-dried specimens. Forty-seven target nuclear genes were recovered from a herbarium specimen of the critically endangered St Helena boxwood, *Mellissia begoniifolia*, collected in 1815. Herbarium specimens yield significantly less high-molecular-weight DNA than silica gel-dried specimens, and genomic DNA quality declines with sample age, which is negatively correlated with target enrichment efficiency. Climate, taxon-specific traits, and collection strategies additionally impact target sequence recovery. We also detected taxonomic bias in targeted sequencing outcomes for the 10 most numerous angiosperm families that were investigated in depth. We recommend that (1) for species distributed in wet tropical climates, silica gel-dried specimens should be used preferentially; (2) for species distributed in seasonally dry tropical climates, herbarium and silica gel-dried specimens yield similar results, and either collection can be used; (3) taxon-specific traits should be explored and established for effective optimisation of taxon-specific studies using herbarium specimens; (4) all herbarium sheets should, in future, be annotated with details of the preservation method used; (5) long-term storage of herbarium specimens should be in stable, low-humidity, and low-temperature environments; and (6) targeted sequencing with universal probes, such as Angiosperms353, should be investigated closely as a new approach for DNA barcoding that will ensure better exploitation of herbarium specimens than traditional Sanger sequencing approaches.

## Introduction

The world’s herbaria collectively house millions of preserved specimens, including many endangered or extinct species and type specimens (Staats et al., 2013). The five largest herbaria alone house ∼36 million specimens (Paris, New York, Kew, Missouri, and St. Petersburg; Index Herbariorum Online: http://sweetgum.nybg.org/science/ih/, accessed on 07/03/2019). Herbarium collections are an extraordinary resource for research on the world’s plant diversity but are largely underutilised in molecular research (Buerki and Baker, 2016). This is predominantly due to problems that arise in DNA extraction, amplification, and PCR-based sequencing methods caused by the low quantity and highly degraded nature of DNA in herbarium specimens. Such degradation occurs as a result of specimen preservation methods and long-term storage conditions (Pyle and Adams, 1989; Savolainen et al., 1995; Adams and Sharma, 2010; Staats et al., 2011; Särkinen et al., 2012; Bakker, 2017).

Developments in high-throughput sequencing (HTS) methods have massively increased the potential of herbarium collections in molecular studies. HTS methods can handle very low input DNA quantities and often rely on short fragmented DNA molecules for short-read sequencing (Staats et al., 2013; Jones and Good, 2015). Several studies have demonstrated that genome skimming (a HTS method) can successfully retrieve hundreds of kilobases of DNA sequence data from herbarium specimens on a routine basis. Plastid genome (plastome) and nuclear ribosomal DNA (nrDNA) sequences have been retrieved with HTS from as little as 500 pg of degraded DNA obtained from herbarium specimens up to 80 years old that span a wide phylogenetic range (Zeng et al., 2018), as well as from up to 100-year-old herbarium specimens of *Sartidia* (Poaceae) (Besnard et al., 2014). Bakker et al. (2016) assembled partial plastome sequences from as little as 24 ng of poor-quality input DNA from herbarium specimens up to 146 years old from a number of angiosperm families, while Zedane et al. (2016) obtained the complete plastome, the nrDNA cluster, and partial sequences of low-copy genes from a 140-year-old specimen of the extinct genus *Hesperelaea* (Oleaceae). Furthermore, a full nuclear genome has been recovered from a 43-year-old herbarium specimen of *Arabidopsis thaliana* (Brassicaceae) (Staats et al., 2013).

HTS of herbarium specimens has primarily focused on reconstructing high-copy number organellar genomes (e.g., plastid) or high-copy number nuclear regions (e.g., rDNA) from low-coverage genome skims (Staats et al., 2013). Increasingly, however, targeted sequencing (sequencing of target-enriched libraries) is being applied to herbarium material to retrieve low-copy nuclear gene sequence data. This is because targeted sequencing is more cost-effective and efficient at recovering these low-copy nuclear orthologues than whole-genome sequencing (Gnirke et al., 2009; Mamanova et al., 2010; Cronn et al., 2012; Jones and Good, 2015; McKain et al., 2018), given that genome size varies ∼2,400-fold in angiosperms and can reach a staggering 1C = 148.8 Gb (Dodsworth et al., 2015). Target enrichment uses DNA or RNA probes (‘baits’) to hybridise and capture specific loci within a genomic library, resulting in those targeted loci being preferentially sequenced (Grover et al., 2012; Johnson et al., 2019). Despite the low quantity and quality of input DNA, evidence for the effectiveness of targeted sequencing from herbarium specimens of varying ages and from different angiosperm families and genera is growing rapidly (Hart et al., 2016; Vatanparast et al., 2018; Villaverde et al., 2018; Couvreur et al., 2019).

The potential of targeted sequencing in herbariomics research is now being realised, unlocking a wealth of opportunities in fields such as phylogenetics, population genetics, conservation genetics, and DNA barcoding (Bieker and Martin, 2018). Nevertheless, a range of factors have been identified that impact input DNA quality and sequencing success such as sample age, specimen preservation method, climate, genome size, and taxonomic traits (e.g., leaf texture and tissue type or leaf chemistry) (Staats et al., 2011; Staats et al., 2013; Bakker et al., 2016; Hart et al., 2016; Weiß et al., 2016; Bakker, 2017; Kuzmina et al., 2017). A systematic understanding of these factors across a broad range of plant families is now required.

**Figure 1:**
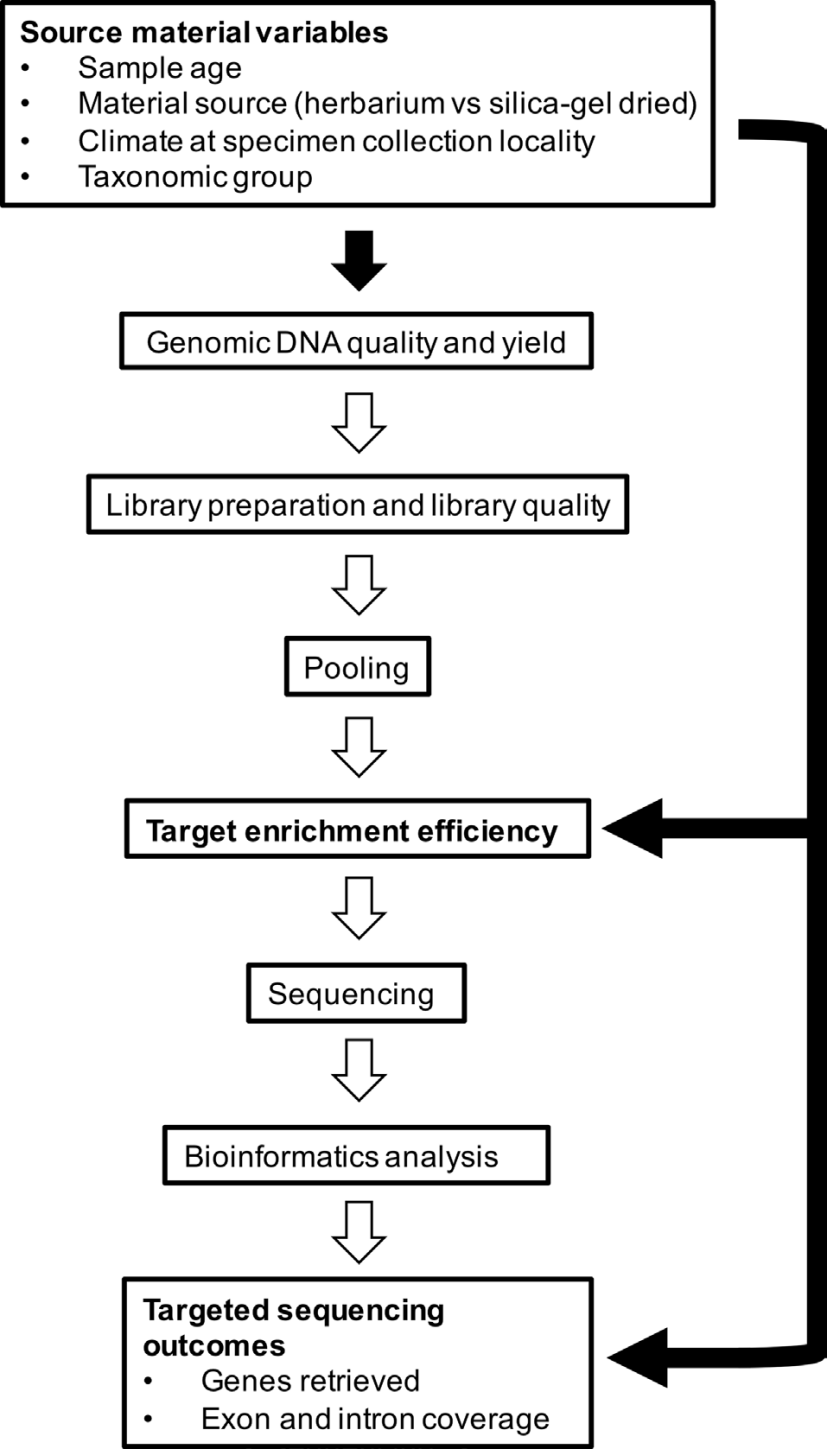
Source material variables impact genomic DNA quality and yield, which feeds into library preparation and quality, pooling, target enrichment efficiency, sequencing, bioinformatics analysis, and targeted sequencing outcomes. In this study, we investigate the relationships shown by the black-filled arrows.

In this study, we aim to investigate factors that affect capture of hundreds of nuclear genes from herbarium specimens that span the diversity of angiosperms. More specifically, we aim to determine whether 1) material source (herbarium versus silica gel-dried), 2) sample age, 3) climate (according to species distributions), and 4) taxonomic group correlate with genomic DNA quality and quantity obtained from extraction and various downstream variables such as target enrichment efficiency, gene retrieval, and mean exon and intron coverage (**Figure 1**). We use a recently developed kit, the Angiosperms353 probe set (Johnson et al., 2019), which was designed to target 353 nuclear genes from any angiosperm family. Our large and diverse dataset was collected as part of the Plant and Fungal Trees of Life project (PAFTOL) (www.paftol.org) at the Royal Botanic Gardens, Kew. We hope that these results will inform the selection of material for targeted sequencing studies, while also influencing curation practices to enhance the potential of herbaria as goldmines for genomic research.

## Materials and Methods

### Sampling and Associated Information

We sequenced 529 specimens belonging to 40 orders, 86 families, 459 genera, and 515 species. The specimens were selected to represent the breadth of angiosperm diversity including all major clades of the Angiosperm Phylogeny Group IV system (The Angiosperm Phylogeny Group et al., 2016). Of those 529 specimens, 435 were sourced from a number of worldwide herbaria and collected between 1815 and 2017, and 94 specimens were sourced from silica gel-dried specimens collected between 1992 and 2017 (**Tables 1** and **S1**). It was not possible to associate a collection year to 100 specimens. These specimens were, therefore, omitted from analyses testing whether sample age correlates with genomic DNA concentration, genomic DNA quality, target enrichment efficiency, gene retrieval, and mean exon and intron coverage.

**Table 1:** Sampling information.

	Herbarium specimens	Silica gel-dried specimens	All specimens
Specimens	435	94	529
Orders	37	17	40
Families	75	27	86
Genera	383	86	459
Species	426	91	515
Collection date range	1815–2017	1992–2017	1815–2017

We used the Plants of the World Online (POWO, 2019) database (http://www.plantsoftheworldonline.org, accessed on 22/03/2019) to assign all species for which climatic information was available to the following categories: desert and/or dry shrubland, seasonally dry tropical, subalpine or subarctic, subtropical, subtropical and tropical, temperate, or wet tropical.

### DNA Extraction, Purification, Quantification, and Quality Evaluation

**Figure 2:**
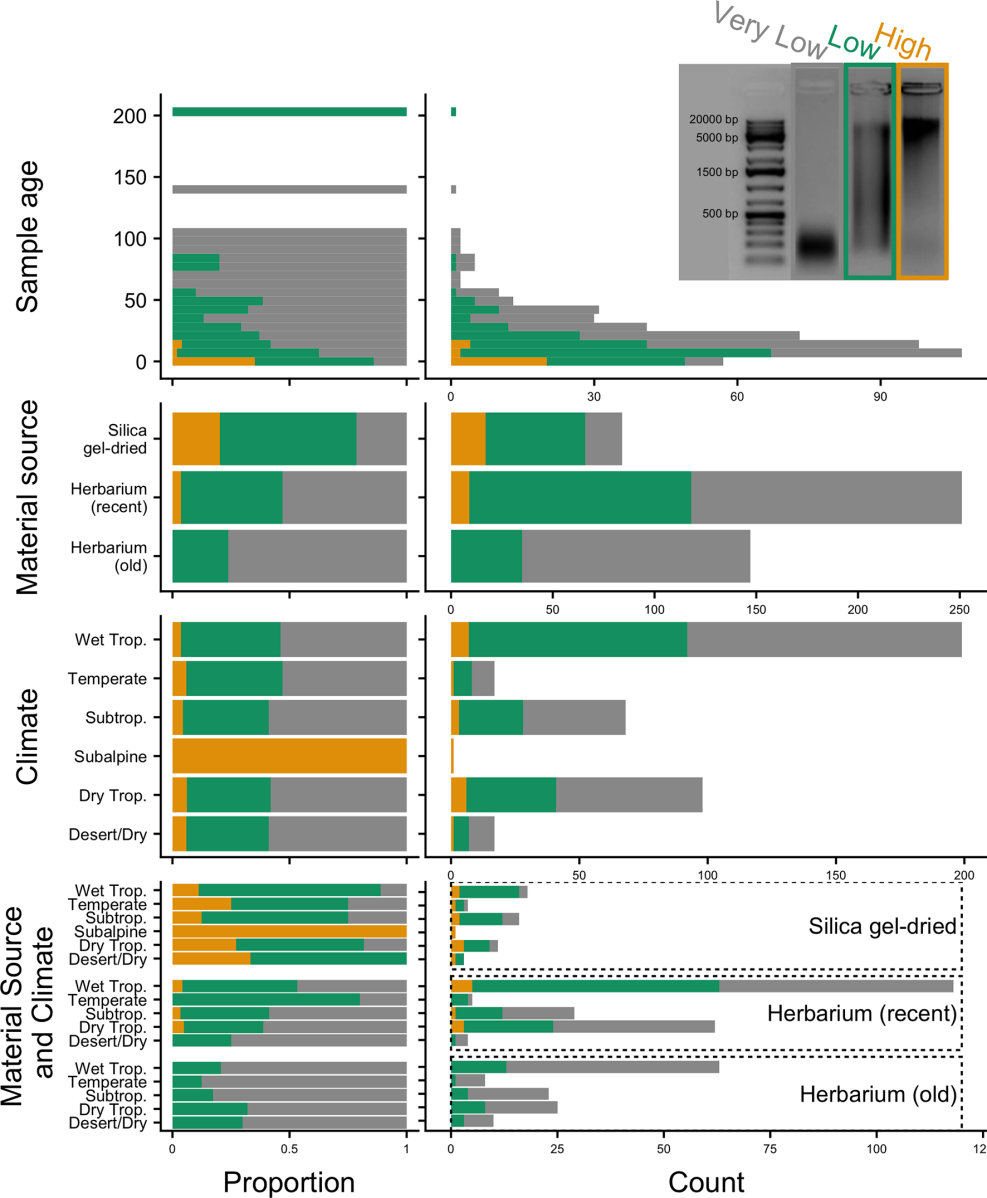
Genomic DNA quality according to sample age, material source, climate (according to species distributions), and material source and climate combined, in relative (proportion) and absolute (count) values. Quality is defined as very low (severely fragmented DNA <500 bp), low (DNA smear on agarose gel), or high (high-molecular-weight DNA >5 kbp).

DNA extractions were performed using a modified cetyltrimethylammonium bromide (CTAB) protocol (Doyle and Doyle, 1987). Approximately 20 mg of leaf tissue was used from silica gel-dried material and 40 mg from herbarium material. Plant tissue was ground in 2-ml tubes with two stainless steel beads using a Mixer Mill MM400 (Retsch GmbH, Germany). We also used existing DNA extractions from the Kew DNA bank obtained using a standard CTAB chloroform, ethanol precipitation, and wash stages, followed by caesium chloride/ethidium bromide density gradient cleaning and dialysis. All DNA extracts were purified using Agencourt AMPure XP Bead Clean-up (Beckman Coulter, Indianapolis, IN, USA), quantified using a Quantus™ Fluorometer (Promega Corporation, Madison, WI, USA), and then run on 1% agarose gel to assess the average fragment size. Samples with very low concentration (not visible on a 1% agarose gel) were assessed on a 4200 TapeStation System using Genomic DNA ScreenTapes (Agilent Technologies, Santa Clara, CA, USA). The quality of the DNA was evaluated based on agarose gel and TapeStation images (e.g., **Figure 2**), with each sample allocated to one of the following DNA quality categories: 1) “high” for samples with high-molecular-weight DNA (>5 kbp), 2) “low” for samples with a DNA smear between high- and low-molecular-weight DNA, and 3) “very low” for samples with severely fragmented, low-molecular-weight DNA (<500 bp).

### Library Preparation, Target Enrichment, and Sequencing

DNA extracts with average fragment sizes above 350 bp were sonicated using an M220 Focused-ultrasonicator^™^ with microTUBES AFA Fiber Pre-Slit Snap-Cap (Covaris, Woburn, MA, USA) following the manufacturer’s protocol and with varied shearing times depending on the DNA fragment size profile, to obtain an average fragment size of 350 bp. Dual-indexed libraries for Illumina^®^ sequencing were prepared using the DNA NEBNext^®^ Ultra™ II Library Prep Kit and the NEBNext^®^ Multiplex Oligos for Illumina^®^ (Dual Index Primers Sets 1 and 2) from New England BioLabs (Ipswich, MA, USA) at either the recommended volumes or half these volumes. The quality of libraries was evaluated on a 4,200 TapeStation System using High Sensitivity D1000 ScreenTapes, and the libraries were quantified using a Quantus Fluorometer. The final average library size including the adapters was ∼500 bp or lower when input DNA fragments were smaller than 350 bp on average.

The libraries were pooled and enriched using the Angiosperms353 probe kit (Arbor Biosciences myBaits^®^ Target Sequence Capture Kit, ‘Angiosperms 353 v1’, Catalogue #308196; Johnson et al., 2019) following the manufacturer’s protocol (v4.0; http://www.arborbiosci.com/mybaits-manual). Hybridisations were performed at 65°C for 24 h in a Hybex^™^ Microsample Incubator (SciGene, CA, USA) and using a volume equivalent to the hybridisation reaction (typically 30 μl) of red Chill-out^™^ Liquid Wax (Bio-Rad, Hercules, CA, USA) to prevent evaporation. Enriched products were amplified with KAPA HiFi 2X HotStart ReadyMix PCR Kit (Roche, Basel, Switzerland) for eight cycles. PCR products were then cleaned using Agencourt AMPure XP Beads. Products were quantified with a Quantus Fluorometer and in some cases re-amplified a second time between three and six cycles. Final products were run on a 4200 TapeStation System using High Sensitivity D1000 ScreenTapes to assess quality and average fragment size. Library pools were multiplexed and sequenced on an Illumina MiSeq with v2 (300 cycles as 2 × 150-bp paired-end reads) or v3 (600 cycles as 2 × 300-bp paired-end reads) chemistry (Illumina, San Diego, CA, USA) at the Royal Botanic Gardens, Kew, or on an Illumina HiSeq producing 2 × 150-bp paired-end reads at Genewiz^®^ (Takeley, UK).

### Data Processing

The reads from the sequencing output files (FASTQ files) were trimmed using Trimmomatic (Bolger et al., 2014) to remove both adapters and reads with a mean Phred quality score <30 and to trim bases from the read endings if their quality was <30 or if they belonged to a 4-bp window with average quality <30, retaining reads with at least 36 bp. Trimmed paired and unpaired reads were processed using HybPiper version 1.3.1 (Johnson et al., 2016) to recover target sequences. The HybPiper pipeline was set to use BLASTX (Camacho et al., 2009) to map the reads to the reference target sequences (available at https://github.com/mossmatters/Angiosperms353; see Johnson et al., 2019, for details on target sequence selection and reference file). Then, each gene was assembled *de novo* using SPAdes (Bankevich et al., 2012), coding sequences were extracted using Exonerate (Slater and Birney, 2005), and non-coding sequences flanking the coding sequences (e.g., introns and UTRs) were recovered using the script intronerate.py, part of HybPiper (Johnson et al., 2016; https://github.com/mossmatters/HybPiper/).

### Raw Data and Exon/Intron Recovery Evaluation

The generated FASTQ files and the HybPiper version 1.3.1 output were evaluated using the get_seq_lengths.py and hybpiper_stats.py scripts also part of HybPiper (Johnson et al., 2016; https://github.com/mossmatters/HybPiper/). For each gene used in the reference target file, the script get_seq_lengths.py calculates the length of the corresponding sequence recovered by HybPiper for each sample. The script hybpiper_stats.py provides for each sample the number of reads; number of reads on target; percentage of reads on target; number of genes with reads; number of genes with contigs; number of genes with sequences; number of genes with sequences >25%, >50%, >75%, and >150% of the target length; and number of genes with paralog warnings. Since we used the BLASTX mapping option of HybPiper, we were not able to retrieve the number of reads and percentage of reads on target with the hybpiper_stats.py script. To obtain these statistics, as well as other statistics such as number of genes with exons, exon coverage, number of genes with introns, and intron coverage for each sample, we combined all the reads that were found by HybPiper to map the reference target files, and we mapped them against the recovered sample gene sequences using BWA (Li and Durbin, 2009; http://bio-bwa.sourceforge.net/bwa.shtml). To produce conservative coverage estimates, we parsed the resulting SAM files using a custom python script to keep only reads mapping with less than three mismatches and a score >30. The filtered SAM files were then analysed with SAMtools (Li et al., 2009) mpileup (Li, 2011) to produce coverage information per base pair, and the outputs were parsed with custom python scripts to calculate intron and exon average coverage for each gene of each sample. Intron–exon boundaries were obtained from the GFF annotation files produced by HybPiper. Scripts are available from the authors.

### Statistical Analyses

We tested whether 1) material source (herbarium versus silica gel-dried), 2) sample age (years between specimen collection and DNA extraction), 3) climate (according to species distributions), and 4) taxonomic group (10 most sampled families: Combretaceae, Connaraceae, Cyperaceae, Fabaceae, Lythraceae, Melastomataceae, Myrtaceae, Rubiaceae, Sapindaceae, and Urticaceae) are statistically correlated with genomic DNA quality (high: >5 kbp, low: DNA smear on agarose gel, and very low:<500 bp; **Figure 2**), genomic DNA concentration (ng/μl; amount of DNA recovered from extraction), target enrichment efficiency (ratio of number of reads mapping to targets and totvwal number of reads), number of genes recovered (with a length ≥50% of the target length), and/or mean exon and intron coverages (**Figure 1**). Given the different time frames in which herbarium and silica gel-dried specimens were collected that could bias our analyses, we categorised all herbarium samples older than 24 years (the age of the oldest silica gel-dried sample) as “old” and those 24 years old or less as “recent.” To avoid biasing statistical analyses by including outliers with excessively large values, data points eight standard deviations higher than the average were removed from downstream analyses. Correlations between continuous variables were tested with Pearson’s correlation tests and evaluated according to their *p*-value, with herbarium samples analysed as a whole and separated into old versus recent. To test for significant differences between groups (old herbarium, recent herbarium, and silica gel-dried), the variable distributions of each group were compared using Kolmogorov–Smirnov (KS) and Mann–Whitney *U* (MW) tests. All the analyses and figures were made in R (R Core Team, 2016) using the packages *cowplot* (Wilke, 2019), *ggplot2* (Wickham, 2009), and *gridExtra* (Auguie, 2017). Scripts are available at github.com/zuntini/herbariomics.

## Results

From the 435 herbarium specimens sequenced, between 6,928 and 22,422,176 (mean: 2,646,149) reads were produced per specimen, of which between 3,870 and 17,780,708 (mean: 2,157,411) were kept after cleaning. The target enrichment efficiency (mapped/total reads) ranged from 0% to 33.5% (mean: 6.1%), and the number of target genes retrieved (defined as those with sequences covering >50% of target length) ranged from 0 to 351 (mean: 231); the exon coverage was between 2.5 and 632.2 times (mean: 44.5 times), while the intron coverage varied between 1.4 and 315.2 times (mean: 29.7 times).

For the 94 silica gel-dried specimens sequenced, the following ranges were observed: total reads 124,858 to 14,347,142 (mean: 3,039,630); cleaned reads 104,225 to 11,894,382 (mean: 2,443,732); target enrichment efficiency 0% to 28.7% (mean: 8.4%); target genes retrieved 2 to 347 (mean: 283.5); exon coverage 2.5 to 296.7 (mean: 48.0 times); and intron coverage 2.3 to 222.7 times (mean: 33.5 times) (**Table S2**).

### Genomic DNA Quality and Concentration

Genomic DNA quality and concentration are critical factors in the early stages of the pipeline leading to target sequence outcomes. The first set of results presented here show the effects of material source, sample age and climate on DNA quality and concentration.

#### Material Source

**Figure 3:**
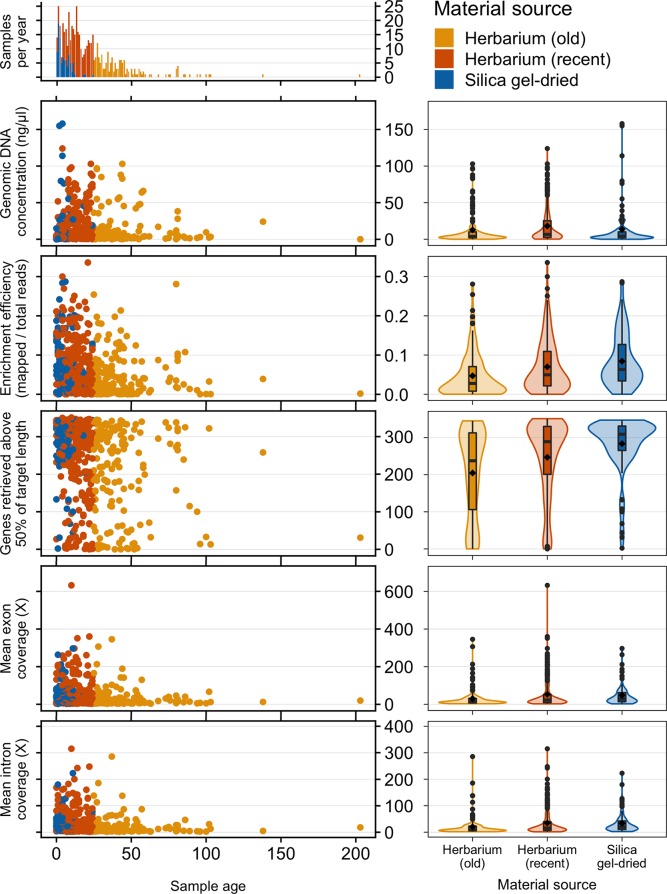
Frequency of samples per age and genomic DNA concentration (ng/μl), target enrichment efficiency (mapped/total reads), genes retrieved above 50% of target length, and mean exon and intron coverage (X) by sample age and material source. Inside each violin plot is a boxplot summarising the interquartile range and median. The diamond symbol denotes the mean while circles represent outliers. The horizontal width of the plot shows the density of the data along the *y*-axis.

Old (more than 24 years old—the age of the oldest silica gel-dried sample) and recent (24 years old or less) herbarium specimens yield predominantly very-low-quality DNA (<500 bp), and old herbarium specimens yield no high-quality DNA (>5 kbp). Silica gel-dried specimens yield more high-quality DNA than recent herbarium specimens (**Figure 2**). There is no difference in mean genomic DNA concentration (ng/μl; obtained from extraction) between silica gel-dried and old herbarium specimens (MW *p*-value: 0.8840; KS *p*-value: 0.9717), but the mean genomic DNA concentration is higher in recent herbarium specimens (MW *p*-values: 0.0049 and 0.0072, respectively, for old herbarium and silica gel-dried specimens; KS *p*-values: 0.0144 and 0.0187, respectively) (**Figure 3**, **Table S3**).

#### Sample Age

The proportion of high-quality DNA declines with age such that no sample older than 23 years yields high-quality DNA. As the sample age increases, the proportion of very-low-quality DNA tends to increase. Exceptions to this trend, such as the single oldest sample (204 years old) yielding low-quality DNA (DNA smear on agarose gel), may be due to very small sample sizes (**Figure 2**). Genomic DNA concentration seems to decrease with sample age, but this correlation is not significant if samples are analysed as a whole or grouped by material source (**Figure 3**, **Table 2**).

**Table 2:** Correlations between sample age and genomic DNA concentration (ng/µl), enrichment efficiency (mapped/total reads), genes retrieved above 50% of target length, and mean exon and intron coverage (X), grouped by material source.

	All samples		Herbarium (old)		Herbarium (recent)		Herbarium (combined)		Silica gel dried	
	corr	*p*-value	corr	*p*-value	corr	*p*-value	corr	*p*-value	corr	*p*-value
Genomic DNA concentration	−0.0514	0.2505	−0.0506	0.5387	0.0619	0.3200	−0.0859	0.0823	0.0540	0.6095
Enrichment efficiency	**−0.2102**	<0.0001	−0.0588	0.4614	**−0.1933**	0.0014	**−0.1861**	0.0001	−0.1619	0.1190
Genes retrieved at 50%	**−0.2412**	<0.0001	−0.0757	0.3387	**−0.1692**	0.0051	**−0.1930**	0.0001	−0.1813	0.0803
Exon coverage	**−0.1449**	0.0009	−0.0792	0.3227	−0.1168	0.0549	**−0.1620**	0.0008	0.0757	0.4684
Intron coverage	**−0.1538**	0.0005	−0.0715	0.3797	−0.1455	0.0172	**−0.1671**	0.0006	0.0601	0.5673

Significant correlation values (p < 0.05) are marked in bold.

#### Climate

The proportion of high-, low-, and very-low-quality DNA does not change when only climate (according to species distributions) is a consideration. However, when the climate is grouped with material source, there does appear to be a variation in the genomic DNA quality. Therefore, climate and material source were grouped in analyses testing for an impact on targeted sequencing variables. The subalpine or subarctic climatic category which only shows high-quality DNA is not a significant result due to the very small sample size of just two silica gel-dried specimens (**Figure 2**). The small sample sizes from desert or dry shrubland, subalpine or subarctic, and temperate climates make it difficult to draw accurate conclusions, so we will focus all further discussion on the tropical climates: wet tropical, subtropical, and seasonally dry tropical.

**Figure 4:**
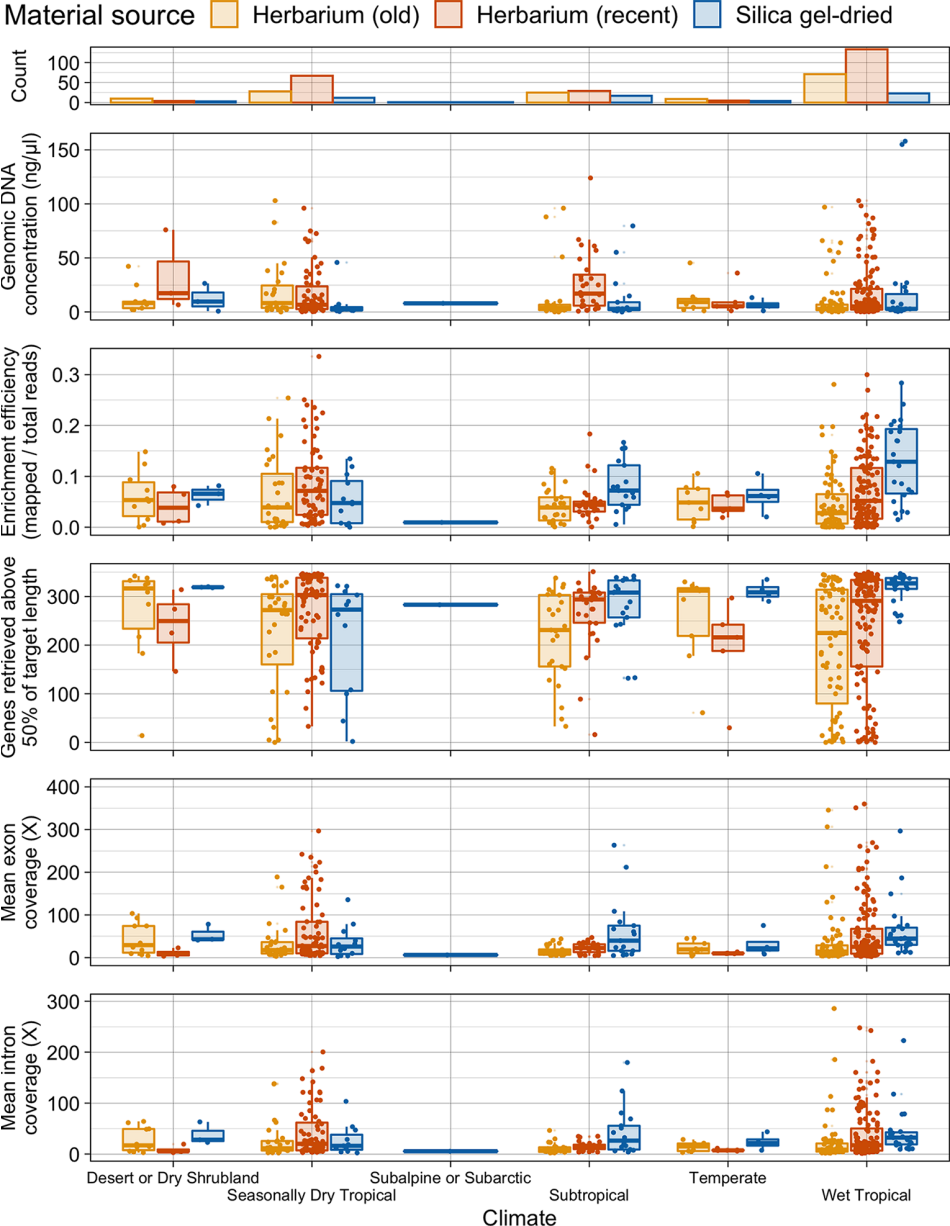
Number of specimens per climate and distribution of genomic DNA concentration (ng/µl), enrichment efficiency (mapped/total reads), genes retrieved above 50% of target length, and mean exon and intron coverage (X) in each climate, grouped by material source. Each boxplot summarises the interquartile range and median.

There is no difference in genomic DNA concentration between old and recent herbarium and silica gel-dried specimens from wet tropical, subtropical, and seasonally dry tropical climates. One exception to the general trend is that genomic DNA concentration is higher in recent herbarium specimens from subtropical climates (**Figure 4**).

### Targeted Sequencing Outcomes

We present the results showing the effects of material source, sample age, climate, and taxonomic groups on targeted sequencing outcomes. We did not test for a correlation between DNA concentration and capture success because we normalised the amount of input DNA for library preparation. The impact of source material variables on targeted sequencing outcomes when grouped by genomic DNA quality shows similar trends to when grouped by material source (see **Figures S1**–**S3**).

#### Material Source

The mean target enrichment efficiency and mean number of genes retrieved are significantly higher in silica gel-dried specimens than in recent herbarium specimens, which in turn are higher than that in old herbarium specimens. The mean exon and intron coverage is higher in silica gel-dried specimens than in old herbarium specimens, but there is no difference in mean exon and intron coverage between silica gel-dried and recent herbarium specimens (MW *p*-values: 0.3016 and 0.0905, and KS *p*-values: 0.2422 and 0.0712, respectively, for exon and intron coverage) (**Figure 3**, **Tables S4**–**S7**).

#### Sample Age

Enrichment efficiency, genes retrieved, and mean exon and intron coverage are negatively correlated to sample age if samples are analysed as a whole and amongst herbarium samples. When grouped by material source and age, recent herbarium specimens are negatively correlated with three variables (except exon coverage), and the only significant correlation for silica gel-dried specimens is between sample age and genes retrieved (**Figure 3**, **Table 2**).

#### Climate

On average, target enrichment efficiency and the number of genes retrieved is higher for silica gel-dried specimens obtained from a wet tropical climate than for silica gel-dried specimens obtained from a subtropical and dry tropical climate and for herbarium (old and recent) specimens obtained from the three tropical climates. A similar pattern is observed in the subtropical climate where silica gel-dried specimens perform best in all four targeted sequencing variables. There is less variation in average target enrichment efficiency, gene retrieval, and exon and intron coverage between herbarium (as a whole) and silica gel-dried specimens obtained from a seasonally dry tropical climate. Recent herbarium specimens obtained from a seasonally dry tropical climate yield the highest results for average target enrichment efficiency and gene retrieval (**Figure 4**).

#### Taxonomic Groups

**Figure 5:**
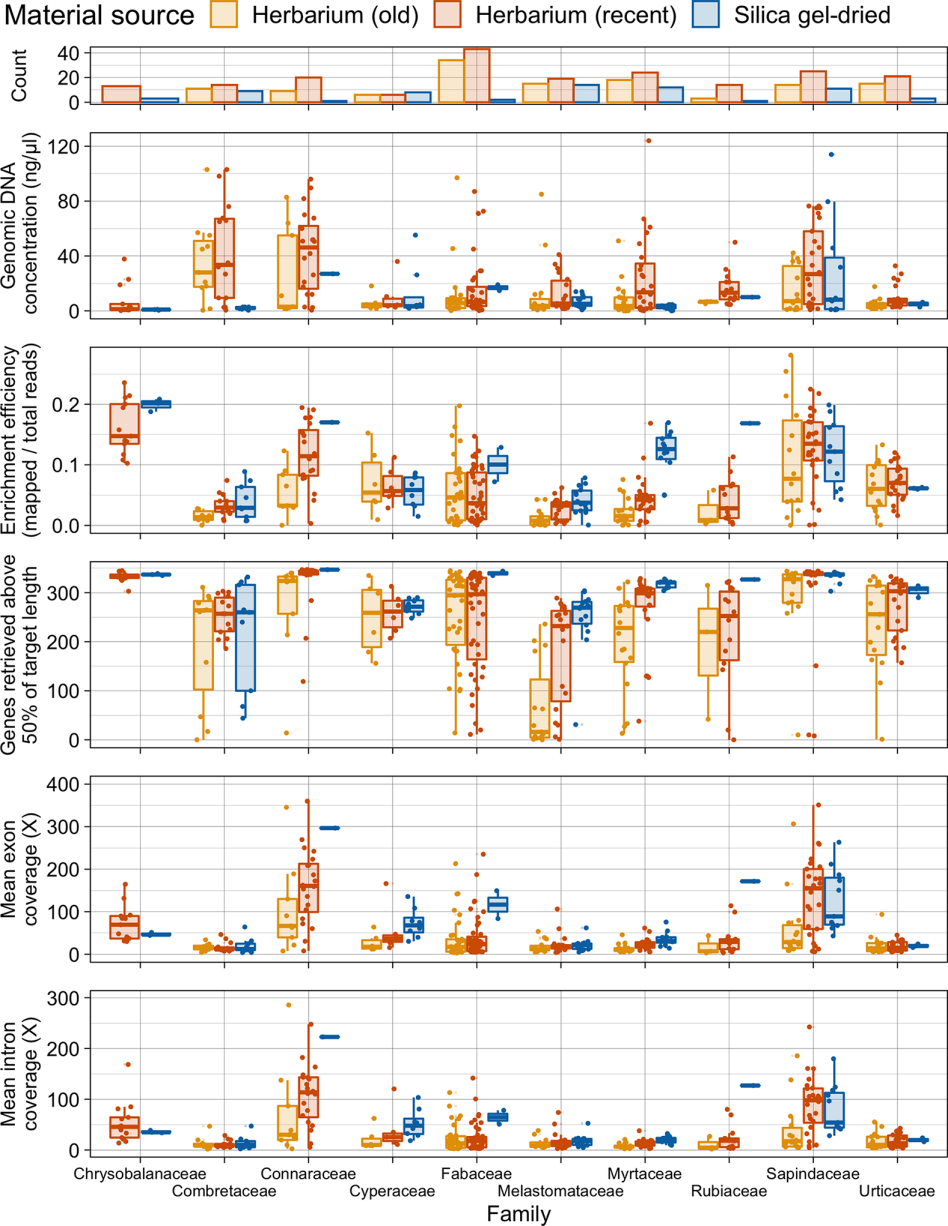
Number of specimens per family and distribution of genomic DNA concentration (ng/µl), enrichment efficiency (mapped/total reads), genes retrieved above 50% of target length, and mean exon and intron coverage (X) in each family, grouped by material source. Each boxplot summarises the interquartile range and median.

Taxonomic bias is evident in the material source dataset (**Figure 5**). All 10 plant families investigated perform slightly differently. In Combretaceae, Connaraceae, Cyperaceae, and Sapindaceae, recent herbarium and silica gel-dried specimens yield similar gene retrieval results. However, in Fabaceae, Lythraceae, Melastomataceae, Myrtaceae, Rubiaceae, and Urticaceae, a general trend of herbarium specimens underperforming, when compared to silica gel-dried specimens, is evident for gene retrieval. Old herbarium specimens generally perform more poorly and produce more variable results compared to other material sources for all families. Exceptions to the general trends are as follows: 1) for Cyperaceae, both old and new herbarium specimens perform as well as silica gel-dried specimens (genes retrieved and target enrichment efficiency); 2) Myrtaceae has a very high target enrichment efficiency for silica gel-dried specimens; 3) in Sapindaceae, the target enrichment efficiency is higher for recent herbarium specimens than for silica gel-dried specimens; and 4) in Urticaceae, both old and new herbarium specimens outperform silica gel-dried specimens for target enrichment efficiency.

## Discussion

We have successfully demonstrated that by using targeted sequencing, we can obtain nuclear gene sequence data from herbarium specimens that span the breadth of angiosperm diversity. Our sample size (529 specimens, 435 from herbaria), breadth of sampling (angiosperm-wide, spanning all major clades), range of sample ages (up to 204 years old), and universal bait kit (designed to target 353 genes from any angiosperm species) make this study the most comprehensive investigation into factors impacting targeted sequencing from herbarium specimens to date. Previous studies have all worked at a much smaller scale and utilised very focused bait sets that are efficient at target enrichment from a single family or genus yet could not be applied across angiosperms (Hart et al., 2016; Vatanparast et al., 2018; Villaverde et al., 2018; Couvreur et al., 2019).

### Genomic DNA Yield and Quality

Genomic DNA quality and yield are important factors that impact various downstream targeted sequencing processes (**Figure 1**). In **Figure S1**, we show that mean target enrichment efficiency, mean number of genes retrieved, and mean exon and intron coverage are positively correlated with genomic DNA quality. The methodology for library preparation (e.g., sonication time, size selection protocol, and number of PCR cycles), as well as library pooling for target enrichment and sequencing, is often modified according to input DNA quality and quantity as in Hart et al. (2016). Since genomic DNA quality and yield are so crucial to targeted sequencing success, it is important to understand what variables affect them. In this study, we investigated the impact of material source (herbarium versus silica gel-dried), sample age, climate (according to species distributions), and taxonomic group on genomic DNA quality and concentration (both measures of DNA extract quality). It was beyond the scope of the dataset used in this study to include genomic DNA yield, library quality, pooling, sequencing, and bioinformatic analysis variables in our analyses.

### Material Source

We found that on average 231 genes were successfully retrieved from herbarium specimens, although the quality of our sequence data in terms of mean target enrichment efficiency and mean number of genes retrieved is significantly higher for silica gel-dried specimens. One of our notable successes was recovering 47 targeted genes from a herbarium specimen of the critically endangered St Helena boxwood, *Mellissia begoniifolia*, collected in 1815.

In agreement with our findings, nuclear gene capture success has previously been reported to be higher from silica gel-dried material than from herbarium material (Villaverde et al., 2018). This was not the case in some studies (Vatanparast et al., 2018; Couvreur et al., 2019), but this is likely due to sample sizes being too small (as little as two herbarium specimens). The variation in targeted sequencing success between herbarium and silica gel-dried specimens could be explained by genomic DNA quality. In this study, herbarium specimens were shown to yield more degraded DNA (<500 bp) compared to silica gel-dried specimens, similar to previous findings (Hart et al., 2016). There was no significant difference in mean genomic DNA concentration between old herbarium and silica gel-dried specimens, but the mean genomic DNA concentration was higher for recent herbarium specimens. This could be due to the CTAB method of DNA extraction used, which has previously been shown to provide the highest DNA yield for herbarium specimens (Särkinen et al., 2012), and therefore, we would not expect material source to affect the amount of DNA obtained using this method. Nonetheless, the degraded nature of DNA in herbarium specimens hinders downstream targeted sequencing processes and is a result of both specimen preparation methods and long-term storage conditions (Pyle and Adams, 1989; Adams and Sharma, 2010; Staats et al., 2011; Särkinen et al., 2012).

### Sample Age

Herbarium specimen storage over time can contribute to DNA degradation (Weiß et al., 2016) and subsequently impact genome sequencing. Adams and Sharma (2010) found that the size of genomic DNA fragments from herbarium specimens decreased with age from large DNA fragments (100K to 1,000 bp) to short DNA fragments (asymptoting at ∼200–500 bp) after 30 years. Furthermore, Hart et al. (2016) found that no herbarium specimens more than 11 years old contained high-molecular-weight DNA. In this study, genomic DNA concentration declines with age, as does the proportion of high-quality DNA, such that no sample older than 23 years yields high-quality DNA. There have been a number of observations of a negative correlation between herbarium sample age and total reads per sample (Bakker et al., 2016) and between herbarium and silica gel-dried sample age and percentage of mapped reads per sample (Villaverde et al., 2018). In the latter, however, this correlation did not hold for the majority of herbarium specimens. Furthermore, a number of studies did not find a significant correlation between the date of specimen collection/sample age and the number of base pairs of conservatively called sequence (one measure of sequence data quality) (Hart et al., 2016) or input DNA yield (Bakker et al., 2016; Zeng et al., 2018). In this study, target enrichment efficiency and mean exon and intron coverage decline with sample age, but there is no relationship between sample age and number of genes retrieved. This suggests that in some cases, other variables aside from sample age (e.g., specimen preparation method used according to climate at specimen collection locality; see below) may be more important in determining genomic DNA quantity and quality and in turn targeted sequencing success.

### Specimen Preparation Method and Climate

In addition to storage in suboptimal conditions over time, DNA degradation occurs as a result of the initial herbarium specimen preparation process. Following field collection, herbarium specimens may be exposed to heat for drying, cold for decontamination, and chemicals for pest control (Schrenk, 1888; Doyle and Dickson, 1987; Bridson and Forman, 2010). Furthermore, many collectors temporarily preserve specimens in alcohol in the field when drying facilities cannot be immediately accessed (i.e., the Schweinfurth method). All of these treatments damage and fragment DNA substantially when the specimen is being prepared for its subsequent storage (Doyle and Dickson, 1987; Pyle and Adams, 1989; Staats et al., 2011; Särkinen et al., 2012).

Given the field conditions in wet tropical environments (e.g., high humidity), specimens may require longer to dry and are thus often treated with alcohol. The impact this has on DNA quality in turn interferes with sequencing success. Herbarium specimens collected in the wet tropics have been found to have higher plastome assembly fragmentation (higher number of contigs per assembly and lower N50 values) and lower sequencing success rates than specimens collected in dry environments (Bakker et al., 2016). In this study, we found that on average, target enrichment efficiency and the number of genes retrieved were highest for silica gel-dried specimens obtained from a wet tropical climate, whereas there was less variation in average target enrichment efficiency, gene retrieval, and exon and intron coverage between herbarium (as a whole) and silica gel-dried specimens obtained from a seasonally dry tropical climate. However, these targeted sequencing outcomes did not correspond with our genomic DNA quality results whereby the proportion of very-low-quality DNA was not higher in herbarium specimens obtained from a wet tropical climate. Neither was there more high-quality DNA for silica gel-dried specimens obtained from a wet tropical climate or herbarium specimens obtained from a seasonally dry tropical climate. There was also no difference in genomic DNA concentration between herbarium and silica gel-dried specimens obtained from wet tropical, subtropical, or seasonally dry tropical climates. Preservation histories cannot be obtained from most herbarium specimens, so firm conclusions cannot be drawn regarding the impact of sample preparation method on DNA quality. However, our results indicate that other variables may be impacting targeted sequencing success here such as taxon-specific traits (e.g., leaf texture and secondary compound chemistry; Särkinen et al., 2012) or a lack of adaptation of collection strategies to the environment (e.g., amount of silica gel added).

### Taxonomic Groups

Due to our wide sampling across angiosperms and the universal probe set used, we were able to investigate how taxonomic biases affect targeted sequencing success. All 10 plant families investigated in detail performed differently (see **Figure 5**). Kuzmina et al. (2017) found that taxon bias affected the sequencing efficiency of the Canadian flora. In our dataset for Combretaceae, Connaraceae, Cyperaceae, and Sapindaceae, recent herbarium specimens and silica gel-dried specimens yield similar gene retrieval results. There could be many reasons for this high success rate including taxon-specific traits (e.g., leaf thickness and lower levels of inhibitory secondary metabolites) and environmental humidity impacting specimen preparation methods. However, in Fabaceae, Lythraceae, Melastomataceae, Myrtaceae, Rubiaceae, and Urticaceae, a general trend of herbarium specimens underperforming when compared to silica gel-dried specimens is evident for gene retrieval. There are exceptions to these general trends. For Cyperaceae, both old and new herbarium specimens perform as well as silica gel-dried specimens (genes retrieved and target enrichment efficiency) probably due to leaf architecture and dry environmental conditions. Myrtaceae has a very high target enrichment efficiency for silica gel-dried specimens, and this may be because data from this family were used to design the bait set (Johnson et al., 2019) and the baits themselves may preferentially include sequences closely related to Myrtaceae. In Sapindaceae, the target enrichment efficiency is higher for recent herbarium specimens than for silica gel-dried specimens probably due to the small number of silica gel-dried specimens included in sampling and may also indicate favourable traits for material preservation. In Urticaceae, both old and new herbarium specimens outperform silica gel-dried specimens for target enrichment efficiency. This is an unexpected result that can probably be attributed to secondary metabolites either decaying with time or being retained in silica gel-dried samples, although rather few silica gel-dried samples were included in our study for the family. However, it still indicates that herbarium samples of Urticaceae performed particularly well.

### DNA Barcoding

HTS technologies now offer new avenues for the authentication of plant material using DNA, or DNA barcoding. However, there has been some debate as to which of the several HTS methods available would be the best approach for DNA barcoding (Hollingsworth et al., 2016). Target enrichment has been proposed as a potential viable option, but the lack of a universal probe set with sufficient phylogenetic breadth has been an obstacle. The Angiosperms353 probe set used here is a potential solution to this issue. Targeted sequence capture has two main advantages compared to the established DNA barcoding method based on the Sanger sequencing of two plastid loci (Hollingsworth et al., 2009). Firstly, it generates vastly more data and therefore potential species discrimination power. Secondly, as we have demonstrated here, the approach works well with poor-quality material. The possibility of sequencing a large set of standard markers, even from small amounts of degraded DNA, could potentially vastly extend the reach and application of DNA barcoding. To develop the Angiosperms353 probe as a barcoding tool, further evidence of its power for species discrimination is required, although evidence is growing that it is highly informative in lower-level phylogenetic studies (e.g., Murphy et al., 2019). Other issues include the need to build a reference dataset and the technical challenges and cost (in both lab and bioinformatics) of targeted sequence capture methods. A targeted sequencing approach to barcoding should also ensure that traditional plant DNA barcoding markers are also captured (Hollingsworth et al., 2016) so that existing reference datasets can still be exploited.

### Conclusions

Furthering our understanding of the factors that impact the success of targeted sequencing will lead to methodological improvements, which will enable the retrieval of genomic sequence data preserved in the world’s herbaria. As already highlighted (Buerki and Baker, 2016; Bieker and Martin, 2018), these techniques promise to unleash the potential of vast historical collections on the fields of environmental, evolutionary, and conservation biology.

We have demonstrated that hundreds of genes can be successfully retrieved using targeted sequencing. This has been achieved despite the negative impacts on DNA quality caused by long-term storage, unfavourable preservation techniques, taxon-specific traits, and/or inconsistencies amongst collection methods. Moreover, due to the universal design of our Angiosperms353 probe kit (Johnson et al., 2019), data were successfully obtained from 86 families spanning the breadth of angiosperm diversity. Nevertheless, a number of factors should be taken into account when selecting specimens for genomic analyses.

Based on our findings, we make the following recommendations:

1. For species from wet tropical climates, silica gel-dried specimens should be preferentially used.
2. For species from seasonally dry tropical climates, either silica gel-dried specimens or herbarium specimens may be used. Our results suggest that both give similar performance.
3. Taxon-specific traits affect targeted sequencing success and should be established for optimisation of taxon-focused studies. For the 10 most numerous angiosperm families investigated in depth here, in some families (e.g., Cyperaceae), we found that old and recent herbarium specimens and silica gel-dried specimens yielded similar targeted sequencing success. In others (e.g., Myrtaceae), we found that silica gel-dried material is a critical step towards effective targeted sequencing.
4. A specimen preservation method should be indicated on herbarium specimens. This information is not routinely included on herbarium specimen labels currently, which hampers the selection of specimens for genomic research.
5. To improve the success of targeted sequencing, herbarium specimens should be stored in stable, low-humidity, and low-temperature environments to limit DNA damage and degradation over time as a result of oxidative and hydrolytic processes.
6. Targeted sequencing using universal probes, such as Angiosperms353, should be investigated further as a new approach for DNA barcoding, offering a tractable HTS alternative that would better exploit herbarium specimens than the traditional Sanger sequencing approach.

Target sequencing of herbarium specimens is an effective and inexpensive technique for unlocking the genomic potential of the world’s natural history collections. As the movement to sequence the genomes of all life gathers pace (Lewin et al., 2018), this approach will become increasingly relevant and complementary, ensuring that all the world’s species, not just those for which high-quality DNA can be sourced, can be accounted for in this genomic revolution, including those which persist only in specimen form that have been lost to extinction at the hands of humankind.

## Data Availability Statement

All datasets generated for this study are included in the manuscript/**Supplementary Files**.

## Author Contributions

GB led the writing of the manuscript with contributions from JC, OM, and AZ. OM curated the data. AZ and OM conducted all data analyses. GB, RC, SD, NE, and LP performed the experiments. SB contributed the computer code. NB, ND, NE, SE, SF, AG, and LP assisted with herbarium sampling. WB and FF acquired funding for the research. WB, FF, PK, and IL supervised the research. VB and WE supported the research. All authors commented on the manuscript.

## Funding

This work was supported by grants from the Calleva Foundation, the Garfield Weston Foundation, and the Sackler Trust to the Royal Botanic Gardens, Kew.

## Conflict of Interest

The authors declare that the research was conducted in the absence of any commercial or financial relationships that could be construed as a potential conflict of interest.

## References

[B1] Adams R. P. Sharma L. N. (2010). DNA from herbarium specimens: I. Correlation of DNA size with specimen age. Phytologia 92, 346–353.

[B2] Auguie B. (2017). gridExtra: miscellaneous functions for ‘grid’ graphics. R package version 2.3. Available at: https://CRAN.R-project.org/package=gridExtra.

[B3] Bakker F. T. (2017). Herbarium genomics: skimming and plastomics from archival specimens. Webbia 72, 35–45. 10.1080/00837792.2017.1313383

[B4] Bakker F. T. Lei D. Yu J. Y. Mohammadin S. Wei Z. de Kerke S. (2016). Herbarium genomics: plastome sequence assembly from a range of herbarium specimens using an iterative organelle genome assembly pipeline. Biol. J. Linn. Soc. 117, 33–43. 10.1111/bij.12642

[B5] Bankevich A. Nurk S. Antipov D. Gurevich A. A. Dvorkin M. Kulikov A. S. (2012). SPAdes: a new genome assembly algorithm and its applications to single-cell sequencing. J. Comput. Biol. 19, 455–477. 10.1089/cmb.2012.0021 22506599 PMC3342519

[B6] Besnard G. Christin P. A. Malé P. J. Lhuillier E. Lauzeral C. Coissac E. (2014). From museums to genomics: old herbarium specimens shed light on a C3 to C4 transition. J. Exp. Bot. 65, 6711–6721. 10.1093/jxb/eru395 25258360

[B7] Bieker V. C. Martin M. D. (2018). Implications and future prospects for evolutionary analyses of DNA in historical herbarium collections. Bot. Lett. 165, 409–418. 10.1080/23818107.2018.1458651

[B8] Bolger A. M. Lohse M. Usadel B. (2014). Trimmomatic: a flexible trimmer for Illumina sequence data. Bioinformatics 30, 2114–2120. 10.1093/bioinformatics/btu170 24695404 PMC4103590

[B9] Buerki S. Baker W. J. (2016). Collections-based research in the genomic era. Biol. J. Linn. Soc. 117 (1), 5–10. 10.1111/bij.12721

[B10] Bridson D. M. Forman L. (2010). The herbarium handbook. 3rd edition. Kew: Royal Botanic Gardens.

[B11] Camacho C. Coulouris G. Avagyan V. Ma N. Papadopoulos J. Bealer K. (2009). BLAST+: architecture and applications. BMC Bioinformatics 10, 421. 10.1186/1471-2105-10-421 20003500 PMC2803857

[B12] Couvreur T. L. P. Helmstetter A. J. Koenen E. J. M. Bethune K. Brandão R. D. Little S. A. (2019). Phylogenomics of the major tropical plant family Annonaceae using targeted enrichment of nuclear genes. Front. Plant Sci. 9, 1941. 10.3389/fpls.2018.01941 30687347 PMC6334231

[B13] Cronn R. Knaus B. Liston A. Maughan P. J. Parks M. Syring J. (2012). Targeted enrichment strategies for next generation plant biology. Am. J. Bot. 99, 291–311. 10.3732/ajb.1100356 22312117

[B14] Dodsworth S. Leitch A. R. Leitch I. J. (2015). Genome size diversity in angiosperms and its influence on gene space. Curr. Opin. Genet. Dev. 35, 73–78. 10.1016/j.gde.2015.10.006 26605684

[B15] Doyle J. J. Dickson E. E. (1987). Preservation of plant samples for DNA restriction endonuclease analysis. Taxon 36, 715–722. 10.2307/1221122

[B16] Doyle J. J. Doyle J. L. (1987). A rapid DNA isolation procedure for small quantities of fresh leaf tissue. Phytochem. Bull. 19, 11–15.

[B17] Gnirke A. Melnikov A. Maguire J. Rogov P. LeProust E. M. Brockman W. (2009). Solution hybrid selection with ultra-long oligonucleotides for massively parallel targeted sequencing. Nat. Biotechnol. 27, 182–189. 10.1038/nbt.1523 19182786 PMC2663421

[B18] Grover C. E. Salmon A. Wendel J. F. (2012). Targeted sequence capture as a powerful tool for evolutionary analysis. Am. J. Bot. 99, 312–319. 10.3732/ajb.1100323 22268225

[B19] Hart M. L. Forrest L. L. Nicholls J. A. Kidner C. A. (2016). Retrieval of hundreds of nuclear loci from herbarium specimens. Taxon. 65, 1081–1092. 10.12705/655.9

[B20] Hollingsworth P. M. Forrest L. L. Spouge J. L. Hajibabaei M. Ratnasingham S. van der Bank M. (2009). A DNA barcode for land plants. Proc. Natl. Acad. Sci. 106 (31), 12794–12797. 10.1073/pnas.0905845106 19666622 PMC2722355

[B21] Hollingsworth P. M. Li D.-Z. van der Bank M. Twyford A. D. (2016). Telling plant species apart with DNA: from barcodes to genomes. Phil. Trans. R. Soc. B 371, 20150338. 10.1098/rstb.2015.0338 27481790 PMC4971190

[B22] Johnson M. G. Gardner E. M. Liu Y. Medina R. Goffinet B. Shaw A. J. (2016). HybPiper: extracting coding sequence and introns for phylogenetics from high-throughput sequencing reads using target enrichment. Appl. Plant Sci. 4, apps.160016. 10.3732/apps.1600016 PMC4948903 27437175

[B23] Johnson M. G. Pokorny L. Dodsworth S. Botigué L. R. Cowan R. S. Devault A. (2019). Universal probe set for targeted sequencing of 353 nuclear genes from any flowering plant designed using k-medoids clustering. Syst. Biol. 68, 594–606. 10.1093/sysbio/syy086 30535394 PMC6568016

[B24] Jones M. R. Good J. M. (2015). Targeted capture in evolutionary and ecological genomics. Mol. Ecol. 25, 185–202. 10.1111/mec.13304 26137993 PMC4823023

[B25] Kuzmina M. L. Braukmann T. W. A. Fazekas A. J. Graham S. W. Dewaard S. L. Rodrigues A. (2017). Using herbarium-derived DNAs to assemble a large-scale DNA barcode library for the vascular plants of Canada. Appl. Plant Sci. 5, apps.170079. 10.3732/apps.1700079 PMC5749818 29299394

[B26] Lewin H. A. Robinson G. E. Kress W. J. Baker W. J. Coddington J. Crandall K. A. (2018). Earth BioGenome Project: sequencing life for the future of life. Proc. Natl. Acad. Sci. 115 (17), 4325–4333. 10.1073/pnas.1720115115 29686065 PMC5924910

[B27] Li H. (2011). A statistical framework for SNP calling, mutation discovery, association mapping and population genetical parameter estimation from sequencing data. Bioinformatics 27, 2987–2993. 10.1093/bioinformatics/btr509 21903627 PMC3198575

[B28] Li H. Durbin R. (2009). Fast and accurate short read alignment with Burrows–Wheeler transform. Bioinformatics 25, 1754–1760. 10.1093/bioinformatics/btp324 19451168 PMC2705234

[B29] Li H. Handsaker B. Wysoker A. Fennell T. Ruan J. Homer N. (2009). The sequence alignment/map (SAM) format and SAMtools. Bioinformatics 25, 2078–2079. 10.1093/bioinformatics/btp352 19505943 PMC2723002

[B30] Mamanova L. Coffey A. J. Scott C. E. Kozarewa I. Turner E. H. Kumar A. (2010). Target-enrichment strategies for next-generation sequencing. Nature Meth. 7, 111–118. 10.1038/nmeth.1419 20111037

[B31] McKain M. R. Johnson M. G. Uribe-Convers S. Eaton D. Yang Y. (2018). Practical considerations for plant phylogenomics. Appl. Plant Sci. 6, e1038. 10.1002/aps3.1038 29732268 PMC5895195

[B32] Murphy B. Forest F. Barraclough T. Rosindell J. Bellot S. Cowan R. (2019). A phylogenomic analysis of *Nepenthes* (Nepenthaceae). bioRxiv, 680488. 10.1101/680488 31682924

[B33] POWO (2019). Plants of the World Online. Facilitated by the Royal Botanic Gardens, Kew Available at: http://www.plantsoftheworldonline.org/(Accessed March 22, 2019).

[B34] Pyle M. M. Adams R. P. (1989). *In situ* preservation of DNA in plant specimens. Taxon. 38, 576–581. 10.2307/1222632

[B35] R Core Team. (2016). R: a language and environment for statistical computing. R Foundation for Statistical Computing, Vienna, Austria. Available at: https://www.R-project.org/.

[B36] Särkinen T. Staats M. Richardson J. E. Cowan R. S. Bakker F. T. (2012). How to open the treasure chest? Optimising DNA extraction from herbarium specimens. PLoS One 7, e43808. 10.1371/journal.pone.0043808 22952770 PMC3429509

[B37] Savolainen V. Cuénoud P. Spichiger R. Martinez M. D. P. Crèvecoeur M. Manen J. (1995). The use of herbarium specimens in DNA phylogenetics: evaluation and improvement. Plant Syst. Evol. 197, 87–98. 10.1007/BF00984634

[B38] Schrenk J. (1888). Schweinfurth’s method of preserving plants for herbaria. Bull. Torrey Bot. Club 15, 292–293. 10.2307/2477483

[B39] Slater G. S. Birney E. (2005). Automated generation of heuristics for biological sequence comparison. BMC Bioinformatics 6, 31. 10.1186/1471-2105-6-31 15713233 PMC553969

[B40] Staats M. Cuenca A. Richardson J. E. Ginkel R. V. van Petersen G. Seberg O. (2011). DNA damage in plant herbarium tissue. PLoS One 6, e28448. 10.1371/journal.pone.0028448 22163018 PMC3230621

[B41] Staats M. Erkens R. H. J. de Vossenberg B. Wieringa J. J. Kraaijeveld K. Stielow B. (2013). Genomic treasure troves: complete genome sequencing of herbarium and insect museum specimens. PLoS One 8, e69189. 10.1371/journal.pone.0069189 23922691 PMC3726723

[B42] The Angiosperm Phylogeny Group Chase M. W. Christenhusz M. J. M. Fay M. F. Byng J. W. Judd W. S. (2016). An update of the Angiosperm Phylogeny Group classification for the orders and families of flowering plants: APG IV. Bot. J. Linn. Soc. 181, 1–20. 10.1111/boj.12385

[B43] Vatanparast M. Powell A. Doyle J. J. Egan A. N. (2018). Targeting legume loci: a comparison of three methods for target enrichment bait design in Leguminosae phylogenomics. Appl. Plant Sci. 6, e1036. 10.1002/aps3.1036 29732266 PMC5895186

[B44] Villaverde T. Pokorny L. Olsson S. Rincón-Barrado M. Johnson M. G. Gardner E. M. (2018). Bridging the micro- and macroevolutionary levels in phylogenomics: Hyb-Seq solves relationships from populations to species and above. New Phytol. 220, 636–650. 10.1111/nph.15312 30016546

[B45] Weiß C. L. Schuenemann V. J. Devos J. Shirsekar G. Reiter E. Gould B. A. (2016). Temporal patterns of damage and decay kinetics of DNA retrieved from plant herbarium specimens. R. Soc. Open Sci. 3, 160239. 10.1098/rsos.160239 27429780 PMC4929915

[B46] Wickham H. (2009). ggplot2: elegant graphics for data analysis. New York: Springer-Verlag. 10.1007/978-0-387-98141-3

[B47] Wilke C. O. (2019). Cowplot: streamlined plot theme and plot annotations for ‘ggplot2’. R package version 094 Available at: https://CRAN.R-project.org/package=cowplot.

[B48] Zedane L. Hong-Wa C. Murienne J. Jeziorski C. Baldwin B. G. Besnard G. (2016). Museomics illuminate the history of an extinct, paleoendemic plant lineage (*Hesperelaea*, Oleaceae) known from an 1875 collection from Guadalupe Island, Mexico. Biol. J. Linn. Soc. 117, 44–57. 10.1111/bij.12509

[B49] Zeng C.-X. Hollingsworth P. M. Yang J. He Z.-S. Zhang Z.-R. Li D.-Z. (2018). Genome skimming herbarium specimens for DNA barcoding and phylogenomics. Plant Methods 14, 43. 10.1186/s13007-018-0300-0 29928291 PMC5987614

