# Supplementary material for: Factors Affecting Targeted Sequencing of 353 Nuclear Genes From Herbarium Specimens Spanning the Diversity of Angiosperms
Source: Front Plant Sci. 2019 Sep 18;10:1102. doi: 10.3389/fpls.2019.01102 (PMC6759688; doi:10.3389/fpls.2019.01102)
Supplement: Supplementary file 1 [file Image_1.pdf]

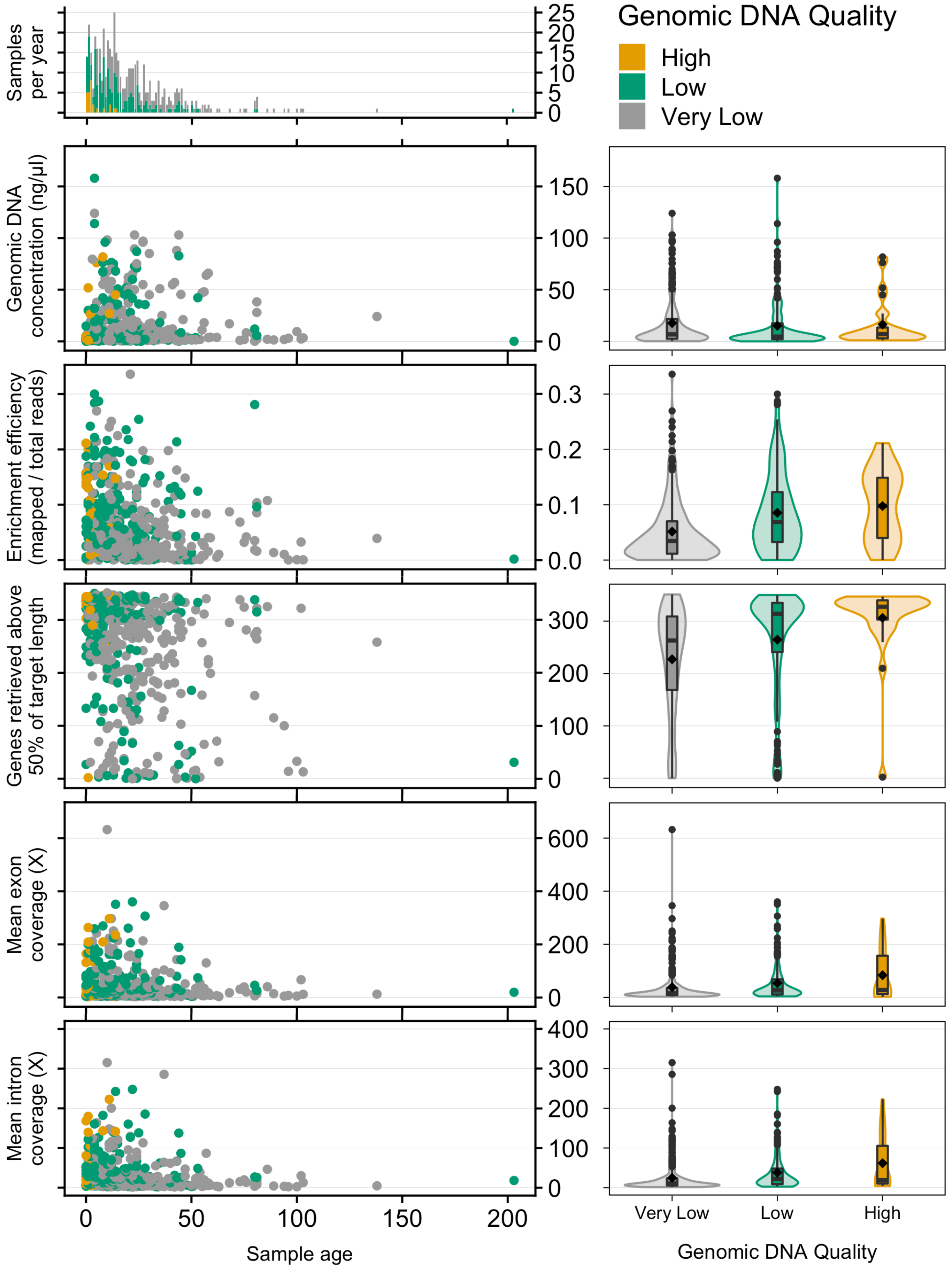

Figure S1 | Frequency of samples per age and genomic DNA concentration (ng/ul), target enrichment efficiency (mapped/total reads), genes retrieved above 50% of target length, and mean exon and intron coverage (X) by sample age and genomic DNA quality. Quality is defined as: very low (severely fragmented DNA <500 bp), low (DNA smear on agarose gel), or high (high molecular weight DNA >5 Kbp). Inside each violin plot is a boxplot summarising the interquartile range and median. The diamond symbol denotes the mean while circles represent outliers. The horizontal width of the plot shows the density of the data along the y-axis.
