# Supplementary material for: Factors Affecting Targeted Sequencing of 353 Nuclear Genes From Herbarium Specimens Spanning the Diversity of Angiosperms
Source: Front Plant Sci. 2019 Sep 18;10:1102. doi: 10.3389/fpls.2019.01102 (PMC6759688; doi:10.3389/fpls.2019.01102)
Supplement: Supplementary file 2 [file Image_2.pdf]

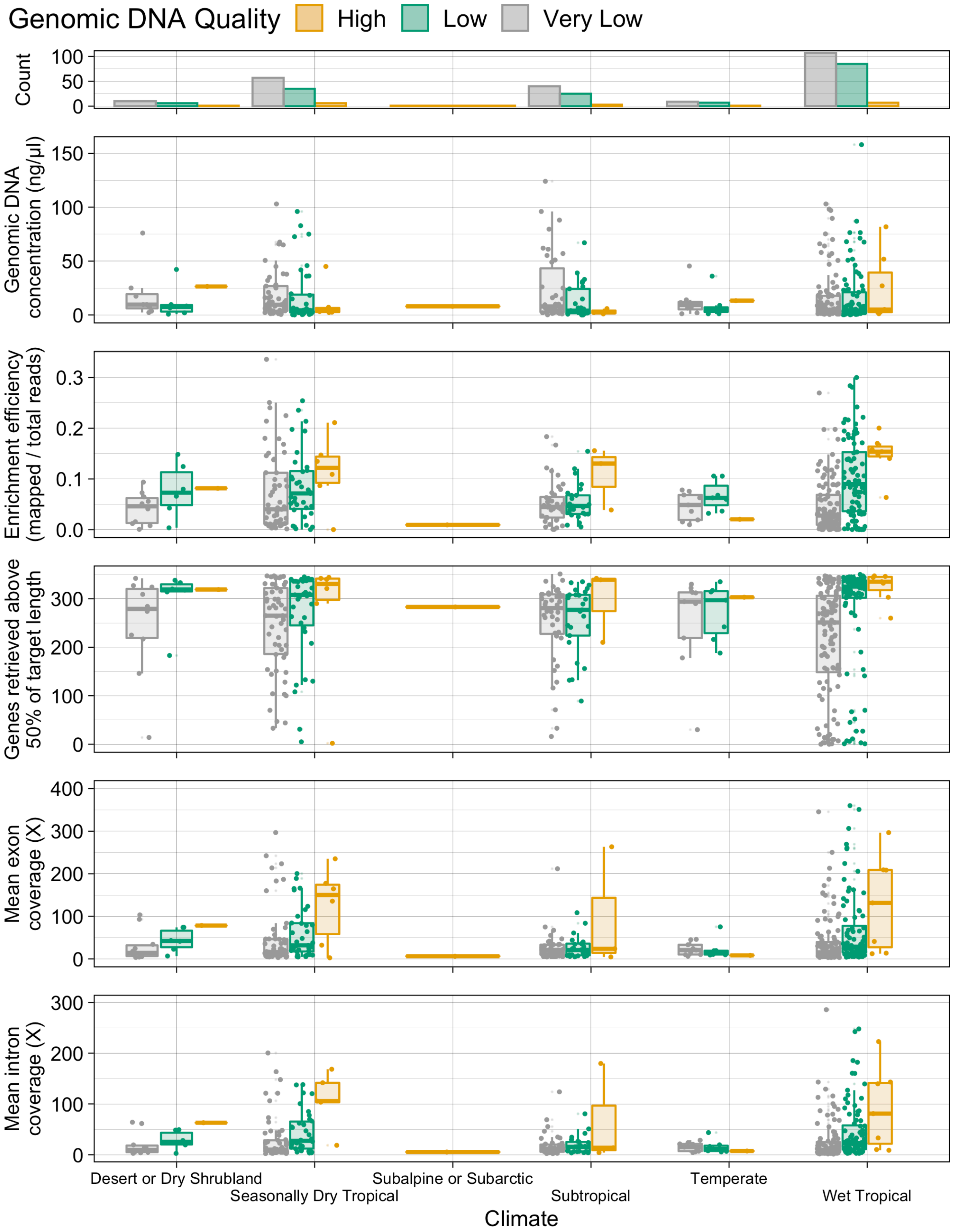

Figure S2 | Number of specimens per climate and distribution of genomic DNA concentration (ng/ul), enrichment efficiency (mapped/total reads), genes retrieved above 50% of target length, and mean exon and intron coverage (X) in each climate, grouped by genomic DNA quality. Quality is defined as: very low (severely fragmented DNA <500 bp), low (DNA smear on agarose gel), or high (high molecular weight DNA >5 Kbp). Each boxplot summarises the interquartile range and median.
